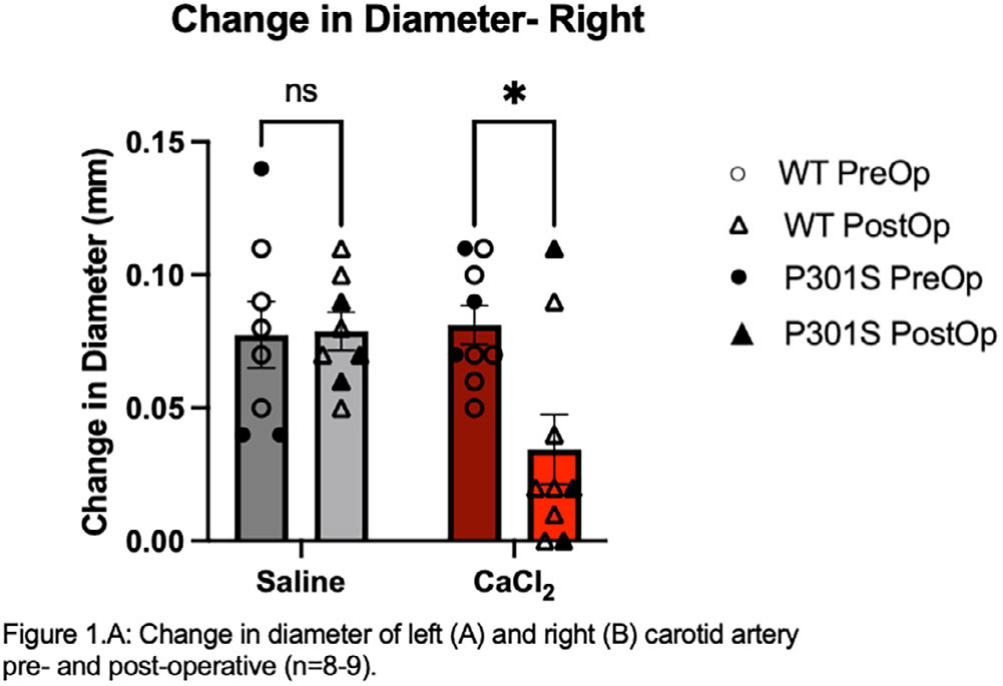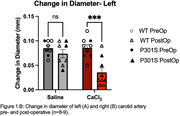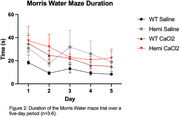# Impact of Surgical Mouse Model of Large Arterial Stiffening on Cognition

**DOI:** 10.1002/alz.092700

**Published:** 2025-01-03

**Authors:** Caroline E Baggeroer, Peter A Pietri, Fiona E. Harrison, Angela L. Jefferson

**Affiliations:** ^1^ Vanderbilt Brain Institute, Vanderbitl University, Nashville, TN USA; ^2^ Vanderbilt Memory & Alzheimer’s Center, Vanderbilt University Medical Center, Nashville, TN USA; ^3^ Vanderbilt University, Nashville, TN USA; ^4^ Department of Medicine, Vanderbilt University Medical Center, Nashville, TN USA; ^5^ Vanderbilt Brain Institute, Vanderbilt University, Nashville, TN USA; ^6^ Vanderbilt Memory and Alzheimer’s Center, Vanderbilt University Medical Center, Nashville, TN USA; ^7^ Department of Neurology, Vanderbilt University Medical Center, Nashville, TN USA; ^8^ Vanderbilt Brain Institute, Vanderbilt University Medical Center, Nashville, TN USA

## Abstract

**Background:**

In humans, larger artery stiffening is associated with increased tau phosphorylation and neurodegeneration. However, because arterial stiffness often co‐occurs with other age‐related conditions like hypertension, atherosclerosis, and diabetes, it is nearly impossible to distill the underlying mechanisms specifically linking arterial stiffening to abnormal brain function. We leveraged a surgical mouse model of larger artery stiffening and used it concurrently with a transgenic Alzheimer’s disease (AD) mouse model of tau pathology to investigate the impact of larger artery stiffening on cognition. Large arterial stiffening typically occurs during middle age, so this model was tested in 5‐month old mice.

**Method:**

5‐month old, male and female, P301S (n = 6) and wild‐type littermate controls (n = 11) underwent a carotid calcification surgery, where both carotid arteries was exposed to 0.3 mol/L CaCl_2_ (or saline as a control) for 20 minutes. Carotid compliance, shown as change in diameter within a cardiac cycle, was measured in vivo using M‐Mode Doppler Ultrasound imaging before and after surgery. Three weeks post‐surgery, mice underwent behavioral testing, including locomotor activity, elevated zero maze, Morris Water maze, Y‐maze, novel object recognition, and nest building.

**Result:**

CaCl_2_ exposure led to decreased post‐operatively compliance of the carotid artery as measured by a significantly decreased change in carotid diameter compared to saline treated mice in both left and right arteries (A. n = 8‐9; p = 0.0003. B. n = 8‐9; p = 0.015) (Figure 1). Preliminary data suggest the carotid calcification surgery impairs ability to learn the position of the escape platform in the Morris water maze in the wild type control mice (Figure 2). Data are not currently sufficiently powered to establish whether the same effect will also be observed in the P301S mice (n = 5‐6 per group WT, 3 per group P301S).

**Conclusion:**

The carotid calcification surgery significantly decreased compliance of the carotid artery in vivo and demonstrated successful stiffening of the large artery. The preliminary data suggest this reduced compliance impairs cognition, specifically spatial learning abilities, but whether the effect is greater in P301S mice is unclear. This model will further be used to investigate whether the carotid calcification surgery accelerates development of tau pathology seen in the P301S mice.